# Cinnamaldehyde inhibits the NLRP3 inflammasome by preserving mitochondrial integrity and augmenting autophagy in *Shigella sonnei*-infected macrophages

**DOI:** 10.1186/s12950-024-00395-w

**Published:** 2024-06-05

**Authors:** Kuo-Feng Hua, Yu-Bei Lin, Hsiao-Wen Chiu, Wei-Ting Wong, Shuk-Man Ka, Chun-Hsien Wu, Wen-Yu Lin, Chien-Chun Wang, Chung-Hua Hsu, Hsien-Ta Hsu, Chen-Lung Ho, Lan-Hui Li

**Affiliations:** 1 Department of Biotechnology and Animal Science, National Ilan University, Ilan, Taiwan; 2 Department of Medical Research, China Medical University Hospital, China Medical University, Taichung, Taiwan; 3 Department of Pathology, Tri-Service General Hospital, National Defense Medical Center, Taipei, Taiwan; 4 Taiwan Autoantibody Biobank Initiative, Hualien Tzu Chi Hospital, Hualien, Taiwan; 5 Graduate Institute of Aerospace and Undersea Medicine, Department of Medicine, National Defense Medical Center, Taipei, Taiwan; 6 Division of Cardiology, Department of Internal Medicine, Tri-Service General Hospital, National Defense Medical Center, Taipei, Taiwan; 7 Infectious Disease Division, Linsen, Chinese Medicine and Kunming Branch, Taipei City Hospital, Taipei, Taiwan; 8 Kunming Prevention and Control Center, Taipei City Hospital, Taipei, Taiwan; 9 Linsen, Chinese Medicine and Kunming Branch, Taipei City Hospital, Taipei, Taiwan; 10 Institute of Traditional Medicine, School of Medicine, National Yang Ming Chiao Tung University, Taipei, Taiwan; 11 Division of Neurosurgery, Taipei Tzu Chi Hospital, Buddhist Tzu Chi Medical Foundation, New Taipei City, Taiwan; 12 School of Medicine, Buddhist Tzu Chi University, Hualien, Taiwan; 13 Division of Wood Cellulose, Taiwan Forestry Research Institute, Taipei, Taiwan; 14 Department of Laboratory Medicine, Linsen, Chinese Medicine and Kunming Branch, Taipei City Hospital, Taipei, Taiwan

**Keywords:** *Shigella sonnei*, NLRP3 inflammasome, Cinnamaldehyde, Pyroptosis, Autophagy

## Abstract

**Background:**

Worldwide, more than 125 million people are infected with *Shigella* each year and develop shigellosis. In our previous study, we provided evidence that *Shigella sonnei* infection triggers activation of the NACHT, LRR, and PYD domain-containing protein 3 (NLRP3) inflammasome in macrophages. NLRP3 inflammasome is responsible for regulating the release of the proinflammatory cytokines interleukin (IL)-1β and IL-18 through the protease caspase-1. Researchers and biotech companies have shown great interest in developing inhibitors of the NLRP3 inflammasome, recognizing it as a promising therapeutic target for several diseases. The leaves of *Cinnamomum osmophloeum* kaneh, an indigenous tree species in Taiwan, are rich in cinnamaldehyde (CA), a compound present in significant amounts. Our aim is to investigate how CA affects the activation of the NLRP3 inflammasome in *S. sonnei*-infected macrophages.

**Methods:**

Macrophages were infected with *S. sonnei*, with or without CA. ELISA and Western blotting were employed to detect protein expression or phosphorylation levels. Flow cytometry was utilized to assess H_2_O_2_ production and mitochondrial damage. Fluorescent microscopy was used to detect cathepsin B activity and mitochondrial ROS production. Additionally, colony-forming units were employed to measure macrophage phagocytosis and bactericidal activity.

**Results:**

CA inhibited the NLRP3 inflammasome in *S. sonnei*-infected macrophages by suppressing caspase-1 activation and reducing IL-1β and IL-18 expression. CA also inhibited pyroptosis by decreasing caspase-11 and Gasdermin D activation. Mechanistically, CA reduced lysosomal damage and enhanced autophagy, while leaving mitochondrial damage, mitogen-activated protein kinase phosphorylation, and NF-κB activation unaffected. Furthermore, CA significantly boosted phagocytosis and the bactericidal activity of macrophages against *S. sonnei*, while reducing secretion of IL-6 and tumour necrosis factor following infection.

**Conclusion:**

CA shows promise as a nutraceutical for mitigating *S. sonnei* infection by diminishing inflammation and enhancing phagocytosis and the bactericidal activity of macrophages against *S. sonnei*.

## Introduction

The NLRP3 inflammasome is a protein complex consisting of NACHT, LRR, and PYD domain-containing protein 3 (NLRP3), apoptosis-associated speck-like protein containing a caspase recruitment domain (ASC) and caspase-1. It regulates the activity of caspase-1 and the maturation and secretion of interleukin (IL)-1β and IL-18. The NLRP3 inflammasome plays important roles in both innate and adaptive immunity [1]. Abnormal activation of the NLRP3 inflammasome is observed in many disease conditions. Inhibition of the NLRP3 inflammasome prevents the pathogenesis of NLRP3-associated diseases, including infections, inflammatory bowel disease, type II diabetes, atherosclerosis, gout, Alzheimer’s disease, arthritis and renal disease [2, 3]. To activate the NLRP3 inflammasome, pattern recognition receptors such as Toll-like receptors must be stimulated. These receptors induce the transcriptional expression of NLRP3 and IL-1β precursor (proIL-1β) through priming signals. The main priming signals include reactive oxygen species (ROS), mitogen-activated protein kinases (MAPKs) and NF-κB [4]. Mere priming signals are not sufficient to activate the NLRP3 inflammasome; additional signals are required to activate the protease caspase-1, which cleaves proIL-1β into IL-1β [5]. The important activation signals include potassium efflux, lysosomal damage and mitochondrial damage [6]. Several negative regulators of the NLRP3 inflammasome have been identified [7], one of which is autophagy. Autophagy eliminates damaged phagolysosomes, endoplasmic reticulum and mitochondria and induces autophagic degradation of ASC and IL-1β [8].

Bacillary dysentery is a food-borne disease caused by *Shigella* infection. Clinical symptoms include diarrhoea, abdominal pain, nausea, vomiting, fever and bloody stool. Each year, *Shigella* infection causes diarrhoea in at least 125 million people worldwide, and the associated mortality rate is approximately 160, 000 people [9]. In developing countries, the prevalent strains of Shigella are *S. dysenteriae*, *S. flexneri* and *S. boydii*, whereas *S. sonnei* is the common strain found in developed countries. Among men who have sex with men, *S. sonnei* can be sexually transmitted [10]. In addition, individuals with human immunodeficiency virus infection are at a higher risk for developing bacillary dysentery [11]. *S. sonnei* is resistant to antibiotics, and the spread of ciprofloxacin-resistant *S. sonnei* has been observed in human immunodeficiency virus-infected populations worldwide [12]. Our previous study provided evidence that *S. sonnei* infection activates the NLRP3 inflammasome in macrophages through mechanisms involving potassium efflux, ROS generation, lysosomal acidification and mitochondrial damage [13].

*Cinnamomum osmophloeum* Kaneh is a tree species native to Taiwan, known for its broad leaves. The essential oil extracted from the leaves of this tree contains cinnamaldehyde (CA) as the main bioactive compound, which has been proven to possess antibacterial, anti-termite and anti-mosquito activities [14–16]. CA exhibits multiple beneficial effects. It not only inhibits xanthine oxidase activity in vitro and reduces the hyperuric acid caused by oxonate in a mouse model [17], but also inhibits the lipopolysaccharide (LPS)-induced inflammatory response in macrophages [18]. Furthermore, CA reduces the expression of vascular cell adhesion protein 1 and intercellular adhesion molecule-1 in tumour necrosis factor (TNF)-stimulated endothelial cells and inhibits the expression levels of cyclooxygenase-2 and prostaglandin E2 in IL-1β-stimulated endothelial cells [19, 20]. Notably, CA exerts anti-inflammatory effects in LPS- and fructose-stimulated mice by inhibiting the NLRP3 inflammasome [21–23]. These findings indicate the potential of CA as an anti-inflammatory agent. However, its effect on NLRP3 inflammasome activation in *S. sonnei*-infected macrophages is unclear.

The NLRP3 inflammasome has become an important target for drug development, with significant implications for the prevention and treatment of NLRP3-associated diseases. In this study, we investigated the inhibitory potential of CA on the NLRP3 inflammasome in *S. sonnei*-infected macrophages. These findings provide a valuable reference for the potential future use of CA as a preventive agent against *S. sonnei*-induced inflammation.

## Materials and methods

### Reagents and chemicals

The trans-Cinnamaldehyde (CA) (CAS number: 14371-10-9; molecular weight: 132.16; ≥ 99%) (239968-50G), *Escherichia coli* O111:B4 LPS (L2630) and gentamicin (G1397) were purchased from Sigma-Aldrich (St. Louis, MO). MCC950 (T3701) was purchased from TargetMol (Wellesley Hills, MA). Antibodies against NLRP3 (AG-20B-0014) and caspase-1 (AG-20B-0044) were purchased from Adipogen International (San Diego, CA). Antibodies against IL-1β (AB-401-NA) were purchased from R&D Systems (Minneapolis, MN). Mouse LC3 CRISPR/Cas9 knockout plasmids (sc-426,563 and sc-417,828-HDR) and antibodies against IL-18 (SC-6177), ASC (SC-22,514-R), cathepsin B (SC-365,558) and actin (SC-47,778) were purchased from Santa Cruz Biotechnology (Santa Cruz, CA). Antibodies against caspase-11 (#14,340), phospho-MAPKs (#9910), IKKβ (#8943), phospho-IKKα/β (#2697), IκBα (#4814), phospho-IκBα (#2859), NF-κB p65 (#4764) and phospho-NF-κB p65 (#3033) were purchased from Cell Signaling Technology (Danvers, MA). MitoTracker Deep Red (M22426), MitoTracker Green (M7514), MitoSOX (M36008), CM-H_2_DCFDA (C6827), ELISA kits of IL-1β (88-7013-88), IL-6 (88-7064-88) and TNF (88-7324-88) were purchased from Thermo Fisher Scientific (Waltham, MA). ELISA kit of IL-10 (431,417) was purchased from Biolegend (San Diego, CA). Antibodies against LC3B (NB100-2220), ATG5 (NB110-53818), p62 (NBP1-48320) and GSDMDC1 (NBP2-33422) were purchased from Novus Biologicals (Littleton, CO). The CytoScan LDH Cytotoxicity Assay kit (786 − 210) was purchased from G-Bioscience (St. Louis, MO). The pNiFty2-SEAP plasmid (pnifty2-seap), QUANTI-Blue solution (rep-qbs3) and Zeocin (ant-zn-05) were purchased from InvivoGen (Carlsbad, CA). Magic Red Cathepsin B assay kit (ICT-937) was purchased from ImmunoChemistry Technologies (San Jose, CA). MitoProbe DiOC_2_(3) assay kit (M34150) for flow cytometry was purchased from Invitrogen (Carlsbad, CA).

### *S. sonnei* strain

*S. sonnei* (strain 25,931) and *Salmonella Typhimurium* (ATCC 14,028) were obtained from the American Type Culture Collection. The culture conditions were described in our previous report [13]. Briefly, *S. sonnei* and *S. Typhimurium* were cultured and subcultured twice weekly on chocolate agar or *Salmonella Shigella* agar, respectively, purchased from Creative Lifesciences (Taipei, Taiwan), at 37 °C in a 5% CO_2_ incubator. For optimal infection efficiency, *S. sonnei* and *S. Typhimurium* were subcultured again one day prior to infection.

### Macrophages cultures

The mouse J774A.1 macrophage cell line and the human THP-1 monocytic cell line were obtained from the Bioresource Collection and Research Center at the Food Industry Research and Development Institute (Hsinchu, Taiwan). THP-1 macrophages were differentiated from the THP-1 monocytic cell line by treatment with 100 nM phorbol 12-myristate 13-acetate for 48 h. Nucleotide-binding domain (NBD) and leucine-rich repeat (LRR) receptor, CARD domain-containing protein 4 (NLRC4) knockout THP-1 monocytic cell line (THP-1 NLRC4-KO) and its parental cells (THP-1-Null2 cells) were procured from InvivoGen (Carlsbad, CA). LC3-knockdown J774A.1 macrophages were generated through transfection with LC3 CRISPR/Cas9 knockout plasmids. The cell clone exhibiting significantly reduced LC3 protein expression in response to CA was selected for further studies. J-Blue cells, an NF-κB reporter cell line, were developed by stable transfection of an NF-κB-inducible reporter plasmid (pNiFty2-SEAP) into J774A.1 macrophages and were selected with Zeocin. Human peripheral blood mononuclear cells (PBMCs) were isolated from the whole blood of healthy volunteers using the Ficoll-Hypaque density gradient centrifugation method. Blood collection was approved by the Institutional Review Board of the Tri-Service General Hospital, National Defense Medical Center, and obtained patients’ informed consent (approval numbers: TSGH-IRB-2-106-05-190 and TSGH-IRB-2-106-05-009). All cells were cultured in RPMI-1640 medium containing 10% fetal bovine serum at 37 °C in a 5% CO_2_ incubator.

### Effect of CA on the activation of inflammasomes induced by *S. Sonnei* or *S. Typhimurium *infection

J774A.1 macrophages, THP-1 NLRC4-KO cells, THP-1-Null2 cells, or PBMCs were primed for 4 h with 1 µg/ml LPS, followed by incubation with CA, 1 µM MCC950, or vehicle (0.1% DMSO) for 0.5 h. The cells were then infected with *S. sonnei* at 50 different multiplicities of infection (MOIs) for 1 h at 37 °C. Afterward, the extracellular bacteria were washed with sterile PBS and cultured in fresh medium containing 3 µg/ml gentamicin and CA or vehicle for an additional 20 h. For *S. typhimurium* infection, J774A.1 macrophages were primed for 4 h with 1 µg/ml LPS, followed by incubation with CA or vehicle for 0.5 h. The cells were infected with *S. typhimurium* at 20 MOIs for 1 h at 37 °C. The expression levels of IL-1β in the culture medium were analyzed by ELISA. The expression levels of proIL-1β/IL-1β, proIL-18/IL-18, caspase-1, caspase-11, Gasdermin D (GSDMD), NLRP3, and ASC in the supernatants were analyzed by Western blotting using methanol/chloroform-concentrated supernatants [24].

### Effect of CA on the expression of TNF, IL-6 and IL-10 induced by *S. Sonnei* infection

J774A.1 macrophages were pre-incubated with CA or vehicle for 24 h, followed by infection with *S. sonnei* at 50 MOIs for 1 h at 37 °C. After removing the extracellular bacteria by washing with sterile PBS, the cells were cultured in fresh medium containing 3 µg/ml gentamicin and CA or vehicle for an additional 20 h. The detectable levels of TNF, IL-6, and IL-10 in the culture medium were analyzed by ELISA.

### LDH release assay

To assess the cytotoxicity of CA, J774A.1 macrophages were treated with 10–40 µM CA, lysis buffer (maximum LDH release), or vehicle (spontaneous LDH release) for 24 h. Additionally, to evaluate the impact of CA on LDH release in *S. sonnei*-infected macrophages, J774A.1 macrophages were primed for 4 h with 1 µg/ml LPS, followed by incubation with CA, 1 µM MCC950, or vehicle for 0.5 h. Subsequently, the cells were infected with *S. sonnei* at 50 MOIs for 1 h at 37 °C. After removing the extracellular bacteria with sterile PBS, the cells were cultured in fresh medium containing 3 µg/ml gentamicin and CA or vehicle for an additional 20 h. Cytotoxicity was determined by measuring LDH levels in the supernatants using the CytoScan LDH Cytotoxicity Assay kit, as previously described [25]. The LDH release percentage was calculated as 100 × (sample OD - spontaneous OD) / (maximum OD - spontaneous OD).

### Effect of CA on the priming signals of the NLRP3 inflammasome induced by LPS

To investigate the impact of CA on the expression of NLRP3 and proIL-1β, J774A.1 macrophages were treated with CA or vehicle for 0.5 h, followed by stimulation with 1 µg/ml LPS for an additional 6 h. The expression levels of NLRP3 and proIL-1β in the cell lysates were analyzed by Western blot. To assess the effect of CA on intracellular ROS production, J774A.1 macrophages were incubated with CA or vehicle for 0.5 h, followed by stimulation with 1 µg/ml LPS for an additional 0.5 h. The cells were then stained with 2 µM CM-H_2_DCFDA for 15 min, and the fluorescence signals were analyzed by flow cytometry. To investigate the impact of CA on the phosphorylation levels of MAPKs and NF-κB-related proteins, J774A.1 macrophages were treated with 40 µM CA or vehicle for 0.5 h, followed by stimulation with 1 µg/ml LPS for an additional 10–60 min. The phosphorylation levels of ERK1/2, JNK1/2, p38, IKKα/β, IκBα, and NF-κB p65 in the cell lysates were analyzed by Western blotting. To examine the effect of CA on the transcriptional activity of NF-κB, J-Blue cells were treated with CA or vehicle for 0.5 h, followed by stimulation with 1 µg/ml LPS for an additional 24 h. The transcriptional activity of NF-κB was analyzed using QUANTI-Blue solution.

### Effect of CA on the mitochondrial damage induced by *S. Sonnei* infection

J774A.1 macrophages and THP-1 macrophages were primed for 4 h with 1 µg/ml LPS, followed by incubation with 40 µM CA or vehicle for 0.5 h. Subsequently, the cells were infected with *S. sonnei* at 50 MOIs for 1 h at 37 °C. After removing the extracellular bacteria with sterile PBS, the cells were cultured in fresh medium containing 3 µg/ml gentamicin and 40 µM CA or vehicle for an additional 20 h. To detect mitochondrial ROS production, the cells were stained with 5 nM MitoSOX for 15 min, and the fluorescence signals were analyzed by flow cytometry and fluorescent microscopy. To assess mitochondrial membrane integrity, the cells were stained with 25 nM MitoTracker Deep Red (for intact mitochondria) and 25 nM MitoTracker Green (for total mitochondria) for 15 min, and the fluorescence signals were analyzed by flow cytometry. To evaluate mitochondrial membrane potential, the cells were stained with 50 nM DiOC_2_(3) for 15 min, and the fluorescence signals were analyzed by flow cytometry.

### Effect of autophagy on CA-mediated IL-1β inhibition in *S. sonnei*-infected macrophages

J774A.1 macrophages were treated with 40 µM CA or vehicle for 4–20 h, or 1 µM rapamycin for 4 h. The expression levels of LC3, ATG5, and p62 in the cell lysates were analyzed by Western blotting. To investigate the impact of CA-mediated autophagy on NLRP3 inflammasome activation in *S. sonnei*-infected macrophages, J774A.1 macrophages were primed for 4 h with 1 µg/ml LPS in the presence or absence of 1 mM 3-MA, followed by incubation with 40 µM CA or vehicle for 0.5 h. The cells were then infected with *S. sonnei* at 50 MOIs for 1 h at 37 °C. After washing the extracellular bacteria with sterile PBS, the cells were cultured in fresh medium containing 3 µg/ml gentamicin and 40 µM CA or vehicle for an additional 20 h. The detectable levels of IL-1β in the culture medium were analyzed by ELISA. Furthermore, wild-type or LC3-knockdown J774A.1 macrophages were incubated with 40 µM CA or vehicle for 20 h. The expression levels of LC3 in the cell lysates were analyzed by Western blot. Additionally, wild-type or LC3-knockdown J774A.1 macrophages were primed for 4 h with 1 µg/ml LPS, followed by incubation with 40 µM CA or vehicle for 0.5 h. The cells were then infected with *S. sonnei* at 50 MOIs for 1 h at 37 °C. After washing the extracellular bacteria with sterile PBS, the cells were cultured in fresh medium containing 3 µg/ml gentamicin and 40 µM CA or vehicle for an additional 20 h. The detectable levels of IL-1β in the culture medium were analyzed by ELISA.

### Effect of CA on the phagocytosis of macrophages against *S. Sonnei*

The study was conducted following our previous methodology with slight adjustments [13]. Briefly, for short-term treatment, J774A.1 macrophages and THP-1 macrophages were treated with 40 µM CA or vehicle for 24 h, followed by infection with *S. sonnei* at 50 MOI for 15 min at 37 °C in a CO_2_ incubator. Afterward, cells were washed with sterile PBS to eliminate extracellular bacteria. Subsequently, cells were exposed to sterile PBS containing 300 µg/ml gentamicin for 1 h at 37 °C in a CO_2_ incubator to eradicate any remaining extracellular bacteria. Following this, cells were again washed with sterile PBS and lysed with 300 µl distilled water. The lysate was then diluted 3000 times (for J774A.1 macrophages) or 10 times (for THP-1 macrophages) with sterile PBS, and 200 µl of the diluted lysate was plated on chocolate agar plates and incubated overnight at 37 °C in a CO_2_ incubator. The colony-forming units (CFUs) were counted and calculated. For long-term treatment, J774A.1 macrophages and THP-1 macrophages were treated with 40 µM CA or vehicle for 24 h, followed by infection with *S. sonnei* at 50 MOI for 15 min at 37 °C in a CO_2_ incubator. Cells were then washed with sterile PBS to remove extracellular bacteria and cultured in medium containing 100 µg/ml gentamicin for an additional 20 h at 37 °C in a CO_2_ incubator. Subsequent steps for lysing cells, dilution, plating, and CFU counting were performed as described above. The final CFU numbers were normalized to the cell counts of each group. Bactericidal activity was represented as the number of killed bacteria, calculated by subtracting the CFU count from the long-term assay from that of the short-term assay.

### Statistical analysis

Two-tailed t tests and ANOVA with Dunnett’s multiple comparisons test were used for statistical analysis for two groups and three or more groups, respectively. Error bars represent the standard deviation of three separate experiments. *, ** and *** represent *p* < 0.05, *p* < 0.01 and *p* < 0.001, respectively.

## Results

### CA inhibits NLRP3 inflammasome activation in *S. sonnei*-infected macrophages

**Fig. 1:**
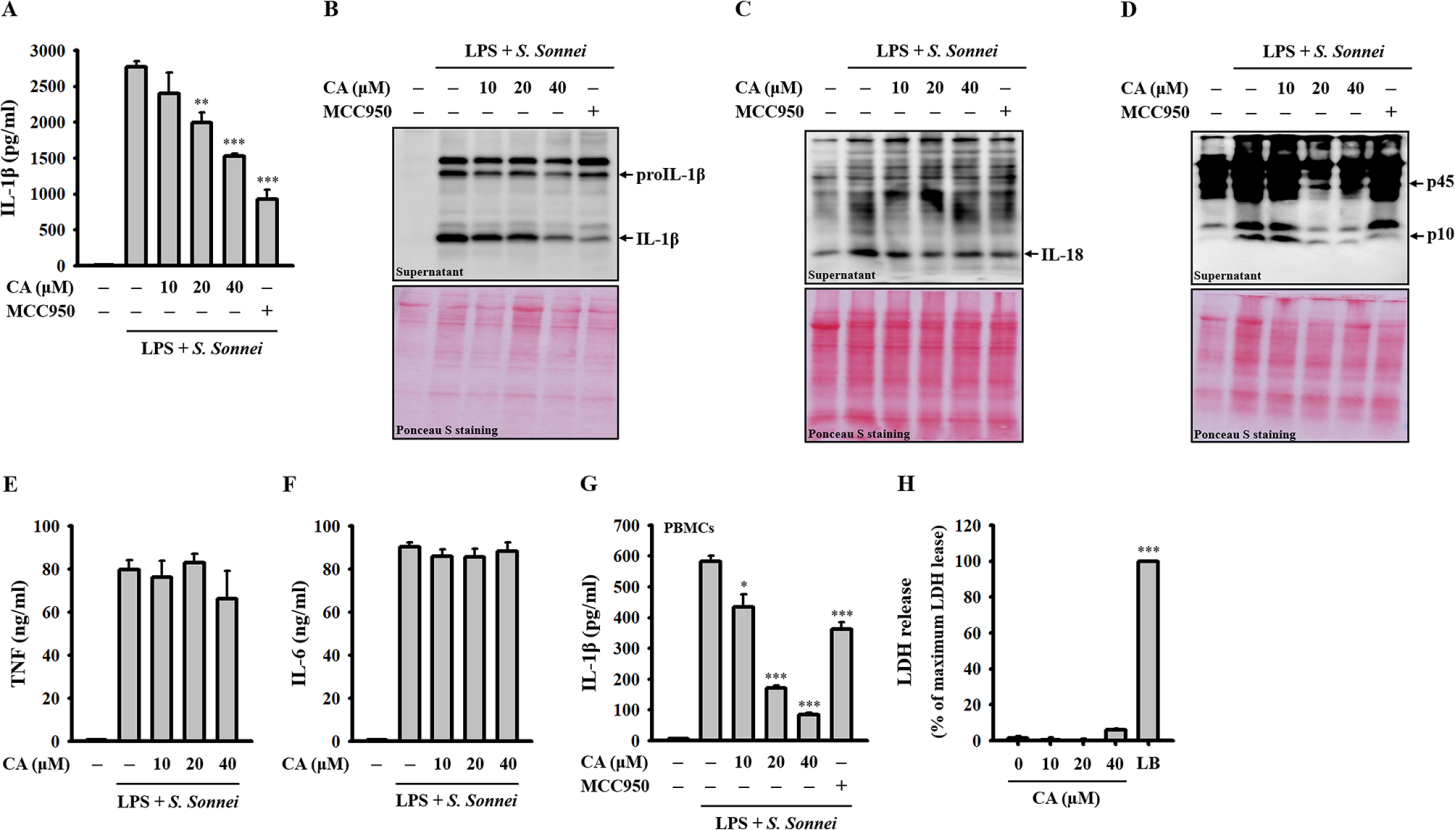
Effect of CA on the NLRP3 inflammasome in *S. sonnei*-infected macrophages. (**A-F**) LPS-primed J774A.1 macrophages were pre-treated with CA or MCC950 for 0.5 h before *S. sonnei* infection for an additional 21 h. The levels of IL-1β in the supernatants were analyzed by ELISA (**A**) and Western blotting (**B**). The detectable levels of IL-18 (**C**), pro-caspase-1 (p45), and active caspase-1 (p10) (**D**) in the supernatants were analyzed by Western blotting. The detectable levels of TNF (**E**) and IL-6 (**F**) in the supernatants were analyzed by ELISA. (**G**) LPS-primed human PBMCs were pre-treated with CA or MCC950 for 0.5 h before *S. sonnei* infection for an additional 21 h. The detectable levels of IL-1β in the supernatants were analyzed by ELISA. (**H**) J774A.1 macrophages were treated with CA for 24 h. The detectable levels of LDH in the supernatants were analyzed by an LDH release assay kit. The ELISA and LDH release data are expressed as the means ± SD of three separate experiments. The Western blotting images are representative of separate experiments. *p < 0.05, **p < 0.01, and ***p < 0.001 compared to the *S. sonnei*-infected macrophages (**A-G**) or control cells (**H**)

To assess the inhibitory effect of CA on the NLRP3 inflammasome in *S. sonnei*-infected macrophages, LPS-primed J774A.1 macrophages were treated with CA or the NLRP3 inhibitor MCC950 for 0.5 h, followed by infection with *S. sonnei* for an additional 21 h. Our findings revealed a dose-dependent reduction in IL-1β secretion upon CA treatment, as determined by ELISA (Fig. 1A) and Western blotting (Fig. 1B). Furthermore, CA treatment resulted in decreased IL-18 secretion, as observed through Western blot analysis (Fig. 1C). Additionally, CA treatment led to a dose-dependent decrease in the levels of active caspase-1 (p10) in the supernatants, as assessed by Western blotting (Fig. 1D). Notably, under the same conditions, CA did not influence the secretion of TNF (Fig. 1E) or IL-6 (Fig. 1F), which are independent of the NLRP3 inflammasome. Furthermore, CA reduced *S. sonnei*-induced IL-1β secretion in LPS-primed human PBMCs (Fig. 1G). Importantly, the observed reduction in NLRP3 inflammasome activation was not attributable to a cytotoxic effect of CA on macrophages, as evidenced by the lack of significant LDH release under the experimental conditions (Fig. 1H). These results collectively suggest that CA selectively inhibits NLRP3 inflammasome activation in *S. sonnei*-infected macrophages.

### CA inhibits pyroptosis in *S. sonnei*-infected macrophages

**Fig. 2:**
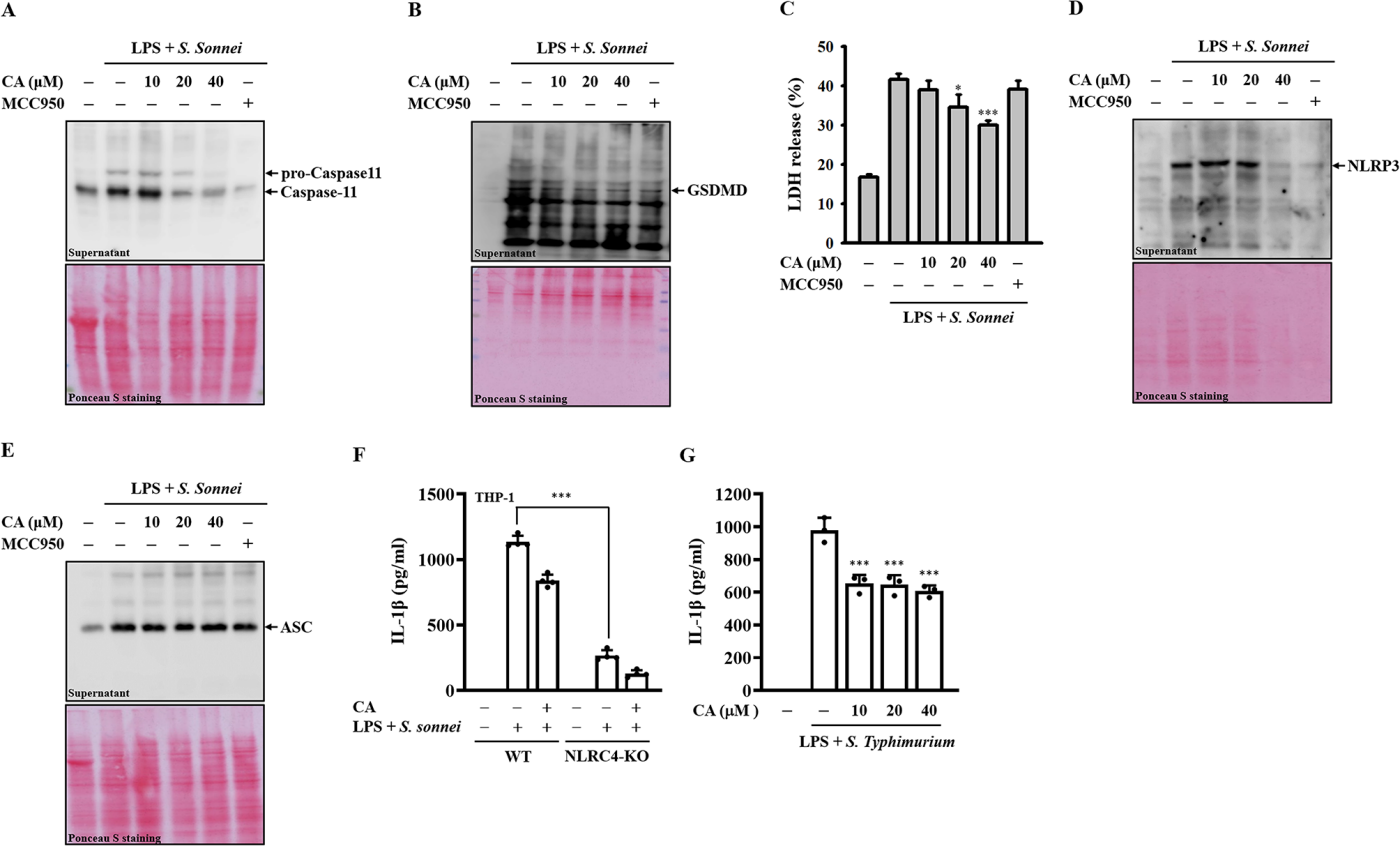
Effect of CA on pyroptosis in *S. sonnei*-infected macrophages. (**A-E**) LPS-primed J774A.1 macrophages were pre-treated with CA or MCC950 for 0.5 h before *S. sonnei* infection for an additional 21 h. The detectable levels of caspase-11 (**A**), GSDMD (**B**), NLRP3 (**D**), and ASC (**E**) in the supernatants were analyzed by Western blotting. The detectable levels of LDH in the supernatants were analyzed by an LDH cytotoxicity assay kit (**C**). (**F**) LPS-primed wild-type or NLRC4-knockout THP-1 macrophages were pre-treated with CA for 0.5 h before *S. sonnei* infection for an additional 21 h. The detectable levels of IL-1β in the supernatants were analyzed by ELISA. (**G**) LPS-primed J774A.1 macrophages were pre-treated with CA for 0.5 h before *S. Typhimurium* infection for an additional 2 h. The Western blotting images are representative of separate experiments. The LDH release and ELISA data are expressed as the means ± SD of three separate experiments. *p < 0.05 and ***p < 0.001 compared to *S. sonnei*-infected macrophages or as indicated

Intracellular release of LPS from infecting bacteria activates noncanonical caspase-11, leading to GSDMD cleavage, plasma membrane pore formation, and pyroptotic cell death [26]. To investigate whether CA could inhibit noncanonical caspase-11 activation in *S. sonnei*-infected macrophages, LPS-primed J774A.1 macrophages were treated with CA for 0.5 h, followed by infection with *S. sonnei* for an additional 21 h. Our results demonstrated that cleaved caspase-11 (Fig. 2A) and GSDMD fragments (Fig. 2B) increased in response to *S. sonnei* infection, and these effects were dose-dependently reduced by CA, as analyzed by Western blotting. Moreover, CA treatment attenuated LDH release caused by *S. sonnei* infection in J774A.1 macrophages (Fig. 2C). Additionally, *S. sonnei* infection induced extracellular release of NLRP3, which was mitigated by CA treatment (Fig. 2D). However, CA did not affect the extracellular release of ASC induced by *S. sonnei* infection (Fig. 2E). Moreover, previous studies have shown that *S. flexneri* infection triggers activation of the NLRC4 inflammasome in macrophages and epithelial cells [27, 28]. Here, we investigated whether the NLRC4 inflammasome is involved in IL-1β production in *S. sonnei*-infected macrophages. To address this, we compared IL-1β production in *S. sonnei*-infected wild-type THP-1 macrophages with that in NLRC4-knockout macrophages derived from the THP-1 human monocytic cell line, achieved through deletion of the critical nucleotide-binding domain region in the *NLRC4* gene. Our findings reveal a significant reduction in IL-1β production in *S. sonnei*-infected NLRC4-knockout macrophages compared to wild-type THP-1 macrophages (Fig. 2F), indicating the contribution of the NLRC4 inflammasome to *S. sonnei*-mediated IL-1β production in macrophages. Notably, we observed a further reduction in IL-1β production in *S. sonnei*-infected NLRC4-knockout macrophages treated with CA (Fig. 2F), suggesting a potential inhibitory effect of CA on the NLRP3 inflammasome. Additionally, CA decreased IL-1β production in J774A.1 macrophages infected with *Salmonella Typhimurium* (Fig. 2G), which activates the NLRC4 inflammasome [29], indicating a possible inhibitory effect of CA on the NLRC4 inflammasome.

### CA does not inhibit the priming signals of the NLRP3 inflammasome in LPS-stimulated macrophages

**Fig. 3:**
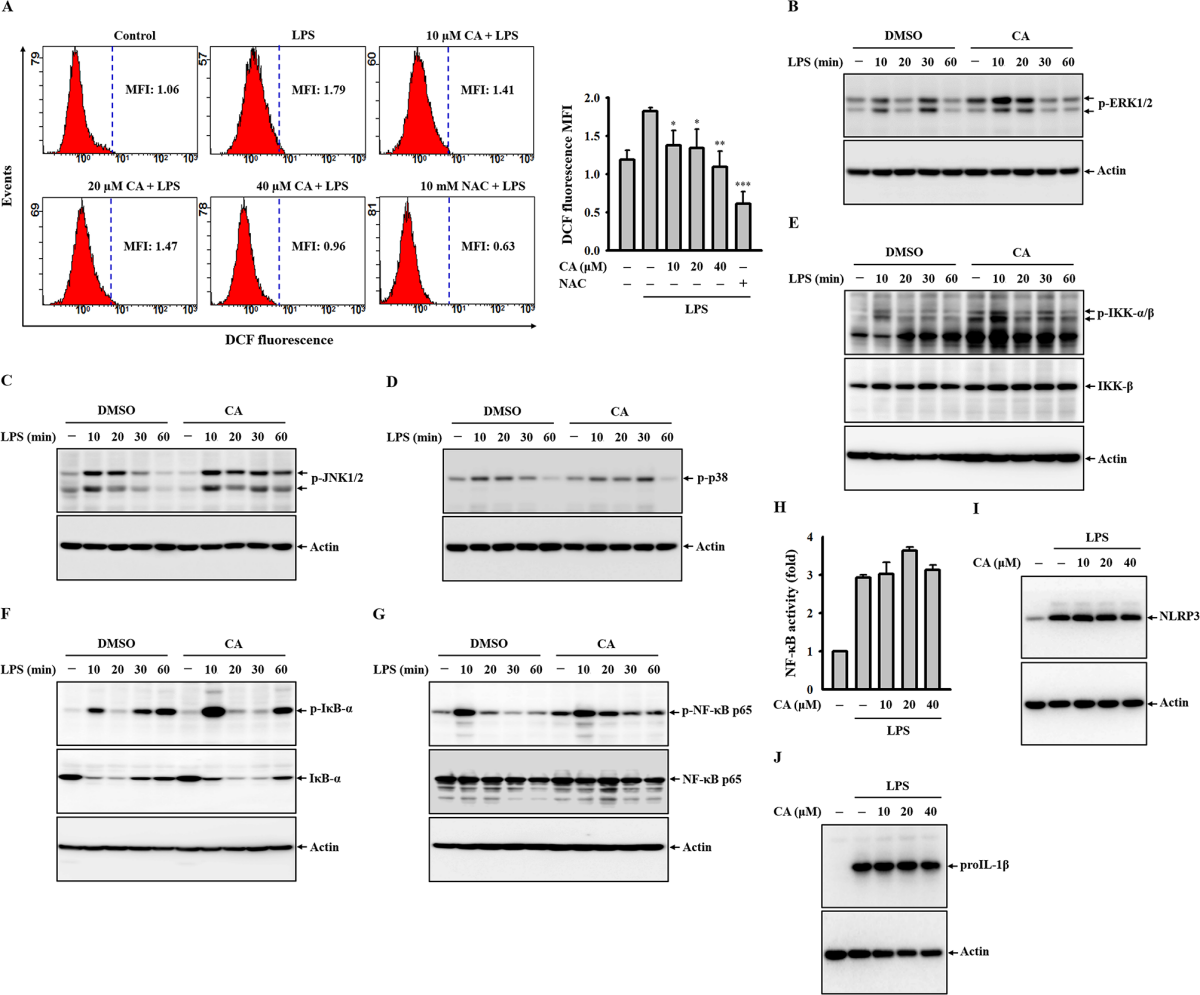
Effect of CA on the priming signals of the NLRP3 inflammasome in LPS-stimulated macrophages. (**A**) J774A.1 macrophages were treated with CA for 0.5 h, followed by incubation with LPS for an additional 0.5 h. Intracellular ROS levels were assessed using CM-H_2_DCFDA staining. (**B-G**) J774A.1 macrophages were pre-treated with 40 µM CA for 0.5 h, then exposed to LPS for 10–60 min. The phosphorylation levels of ERK1/2 (**B**), JNK1/2 (**C**), p38 (**D**), IKK-α/β (**E**), IκB-α (**F**), and NF-κB p65 (**G**) in cell lysates were evaluated by Western blotting. (**H**) J-Blue cells were incubated with CA for 0.5 h, followed by LPS exposure for an additional 24 h. NF-κB transcriptional activity was determined using an NF-κB reporter assay. (**I, J**) J774A.1 macrophages were exposed to CA for 0.5 h, followed by LPS treatment for an additional 6 h. Expression levels of NLRP3 (**I**) and proIL-1β (**J**) in cell lysates were analyzed by Western blotting. ROS and NF-κB activity data represent means ± SD of three independent experiments. Western blot images are representative of distinct experiments. *p < 0.05, **p < 0.01, and ***p < 0.001 compared to LPS-stimulated macrophages

The priming signal for the NLRP3 inflammasome, facilitated by LPS, triggers the transcriptional upregulation of NLRP3 and the precursor of IL-1β (proIL-1β) in macrophages. This process involves activating ROS, MAPKs, or NF-κB pathways [4]. To delve into the mechanisms behind CA’s inhibitory effect on the NLRP3 inflammasome, we incubated J774A.1 macrophages with CA for 0.5 h, followed by LPS for an additional 0.5 h. We then analyzed intracellular ROS levels using CM-H_2_DCFDA staining, observing a dose-dependent reduction in ROS production due to CA (Fig. 3A). To examine CA’s impact on MAPKs activation in LPS-stimulated macrophages, we similarly incubated J774A.1 macrophages with CA for 0.5 h, followed by LPS for 10–60 min. Surprisingly, we found no significant effect on the phosphorylation levels of ERK1/2 (Fig. 3B), JNK1/2 (Fig. 3C), or p38 (Fig. 3D), indicating that CA did not inhibit MAPKs activation. Further investigation into CA’s effect on NF-κB activation in LPS-stimulated macrophages under the same conditions as the MAPKs phosphorylation study revealed no significant impact on the phosphorylation levels of IKK-α/β (Fig. 3E), IκB-α (Fig. 3F), or NF-κB p65 (Fig. 3G). Moreover, CA did not inhibit the transcriptional activity of NF-κB, as evidenced by an NF-κB reporter assay in the J-Blue cell line (Fig. 3H). Finally, the expression levels of NLRP3 (Fig. 3I) and proIL-1β (Fig. 3J) in LPS-stimulated J774A.1 macrophages remained unchanged despite CA treatment. These results collectively suggest that CA does not hinder the NLRP3 inflammasome by impeding priming signals.

### CA reduces cathepsin B release in *S. sonnei*-infected macrophages

**Fig. 4:**
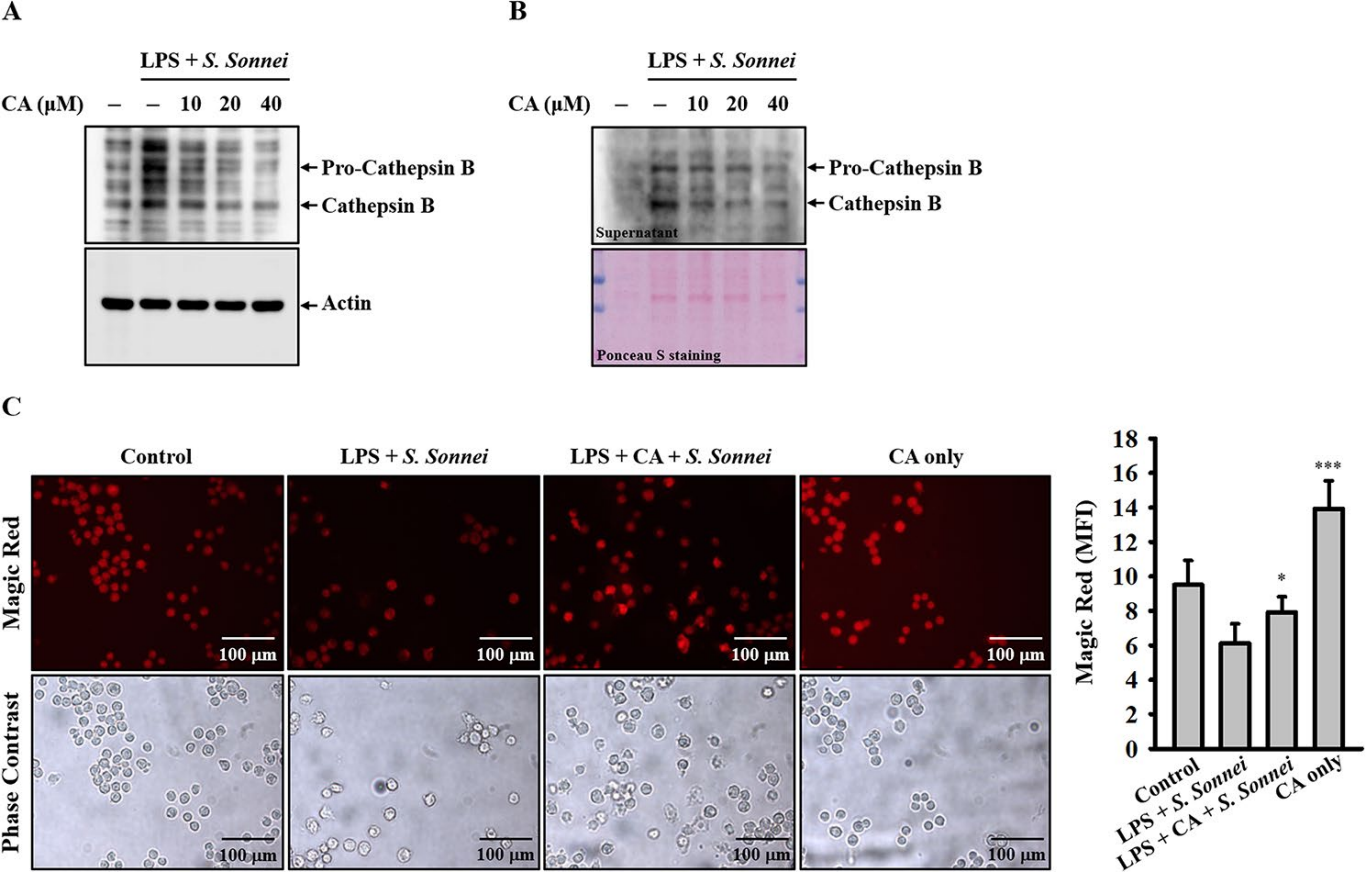
Effect of CA on cathepsin B release in *S. sonnei*-infected macrophages. (**A**) LPS-primed J774A.1 macrophages were incubated with CA for 0.5 h before *S. sonnei* infection for an additional 21 h. The detectable levels of pro-cathepsin B and mature cathepsin B in the cell lysates (**A**) and in the supernatants (**B**) were analysed by Western blotting. The cathepsin B activity was analysed by Magic Red staining and acquired by fluorescent microscopy (**C**). The Western blotting images are representative of separate experiments. The fluorescent microscopy images are representative of different fields, while the histogram providing quantification of mean fluorescent intensity (MFI) expressed as the mean ± SD for these three fields. *p < 0.05 and ***p < 0.001 compared to *S. sonnei*-infected macrophages

Previous research has shown that cathepsin B, released from compromised lysosomes, interacts with NLRP3 in the cytosol, leading to the activation of the NLRP3 inflammasome [30]. In our prior study, we demonstrated that inhibiting lysosomal damage using NH_4_Cl and CQ, both of which inhibit endosomal/lysosomal acidification, significantly reduced IL-1β production in *S. sonnei*-infected macrophages [13]. Additionally, inhibition of cathepsin B by the specific inhibitor CA-074-me also decreased IL-1β production in *S. sonnei*-infected macrophages [13]. These findings underscore the critical roles of lysosomal damage and cathepsin B release in *S. sonnei*-mediated IL-1β production in macrophages. We investigated whether CA inhibits IL-1β production in *S. sonnei*-infected macrophages by mitigating lysosomal damage. To test this hypothesis, we examined the effect of CA on cathepsin B release induced by *S. sonnei* infection in J774A.1 macrophages. Our results indicate that *S. sonnei* infection elevates levels of mature cathepsin B in the cytosol (Fig. 4A) and in supernatants (Fig. 4B), and these effects are dose-dependently reduced by CA (Fig. 4A and B). When cathepsin B is released into the cytosol, its activity is diminished due to the higher pH environment, which inhibits cathepsin B activity. To corroborate the effect of CA on cathepsin B release, we analyzed cathepsin B activity using Magic Red, a substrate for cathepsin B. Our findings reveal that *S. sonnei* infection significantly reduces the fluorescent signal of Magic Red, and this effect is reversed by CA (Fig. 4C). These results indicate that CA attenuates the leakage of cathepsin B into the cytoplasm.

### Effect of CA on mitochondrial damage in *S. sonnei*-infected macrophages

**Fig. 5:**
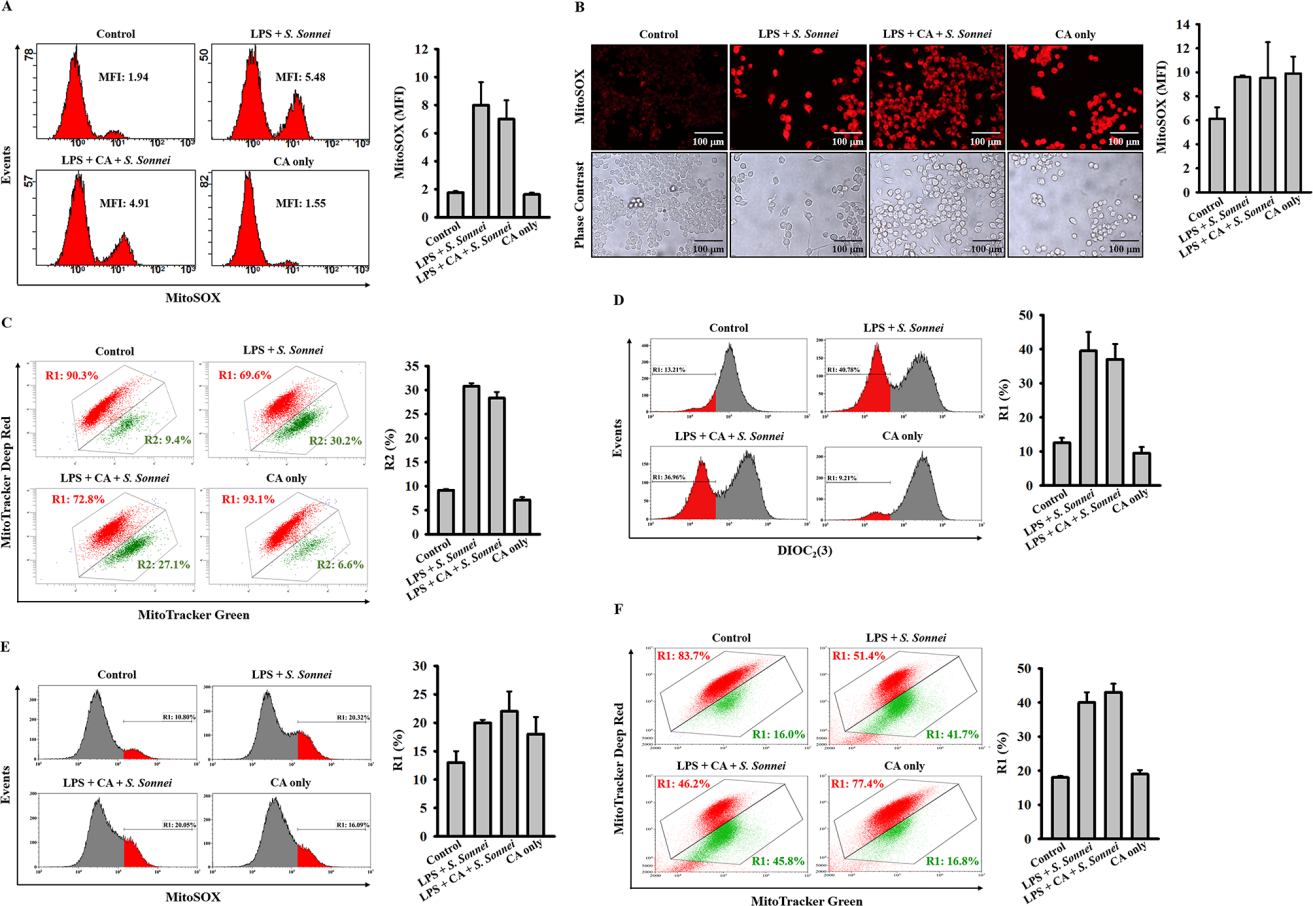
Effect of CA on mitochondrial damage in *S. sonnei*-infected macrophages. (**A-D**) LPS-primed J774A.1 macrophages were treated with 40 µM CA for 0.5 h prior to *S. sonnei* infection for an additional 21 h. Mitochondrial ROS production was assessed by staining with MitoSOX and analysis via flow cytometry (**A**) and fluorescent microscopy (**B**). Mitochondrial membrane integrity was evaluated by staining with MitoTracker Deep Red and MitoTracker Green and analyzed via flow cytometry (**C**). Mitochondrial membrane potential was measured by staining with DIOC_2_(3) and analysis via flow cytometry (**D**). (**E**, **F**) LPS-primed THP-1 macrophages were exposed to 40 µM CA for 0.5 h before *S. sonnei* infection for an additional 21 h. Mitochondrial ROS production was assessed by staining with MitoSOX and analyzed via flow cytometry (**E**), while mitochondrial membrane integrity was evaluated by staining with MitoTracker Deep Red and MitoTracker Green and analyzed via flow cytometry (**F**). The presented images represent individual experiments or fields, and the histogram provides quantification expressed as the mean ± SD for these three experiments or fieldss

We previously demonstrated significant involvement of mitochondrial damage in activating the NLRP3 inflammasome in macrophages during *S. sonnei* infection [13]. Consequently, we investigated whether CA could inhibit the mitochondrial damage induced by *S. sonnei* infection. LPS-primed J774A.1 macrophages were treated with CA for 0.5 hours, followed by infection with *S. sonnei* for an additional 21 hours. We observed that *S. sonnei* infection induced mitochondrial ROS production in macrophages, as indicated by staining with the mitochondrial ROS-specific fluorescent dye MitoSOX and analysis via flow cytometry (Fig. 5A) and fluorescent microscopy (Fig. 5B). However, these effects were not significantly reduced by CA (Fig. 5A and B). Additionally, *S. sonnei* infection caused mitochondrial membrane integrity loss, as shown by staining with the mitochondrial membrane integrity fluorescent dye MitoTracker Deep Red and analyzed via flow cytometry, and this effect was also unaffected by CA (Fig. 5C). Further experiments were conducted to explore the effect of CA on mitochondrial damage using 3,3’-Diethyloxacarbocyanine (DiOC_2_(3)) iodide, a cationic fluorescent dye used to monitor mitochondrial membrane potential. We found that *S. sonnei* infection led to mitochondrial membrane potential loss, which was not impacted by CA (Fig. 5D). Moreover, *S. sonnei* infection induced mitochondrial ROS production (Fig. 5E) and mitochondrial membrane integrity loss (Fig. 5F) in human THP-1 macrophages. Consistent with the results observed in J774A.1 macrophages, CA did not significantly reduce mitochondrial ROS production and mitochondrial membrane integrity loss in *S. sonnei*-infected THP-1 macrophages (Fig. 5E and F). These findings indicate that the inhibition of the NLRP3 inflammasome in *S. sonnei*-infected macrophages by CA is not associated with reducing mitochondrial damage.

### CA inhibits the NLRP3 inflammasome partially through autophagy induction in *S. sonnei*-infected macrophages

**Fig. 6:**
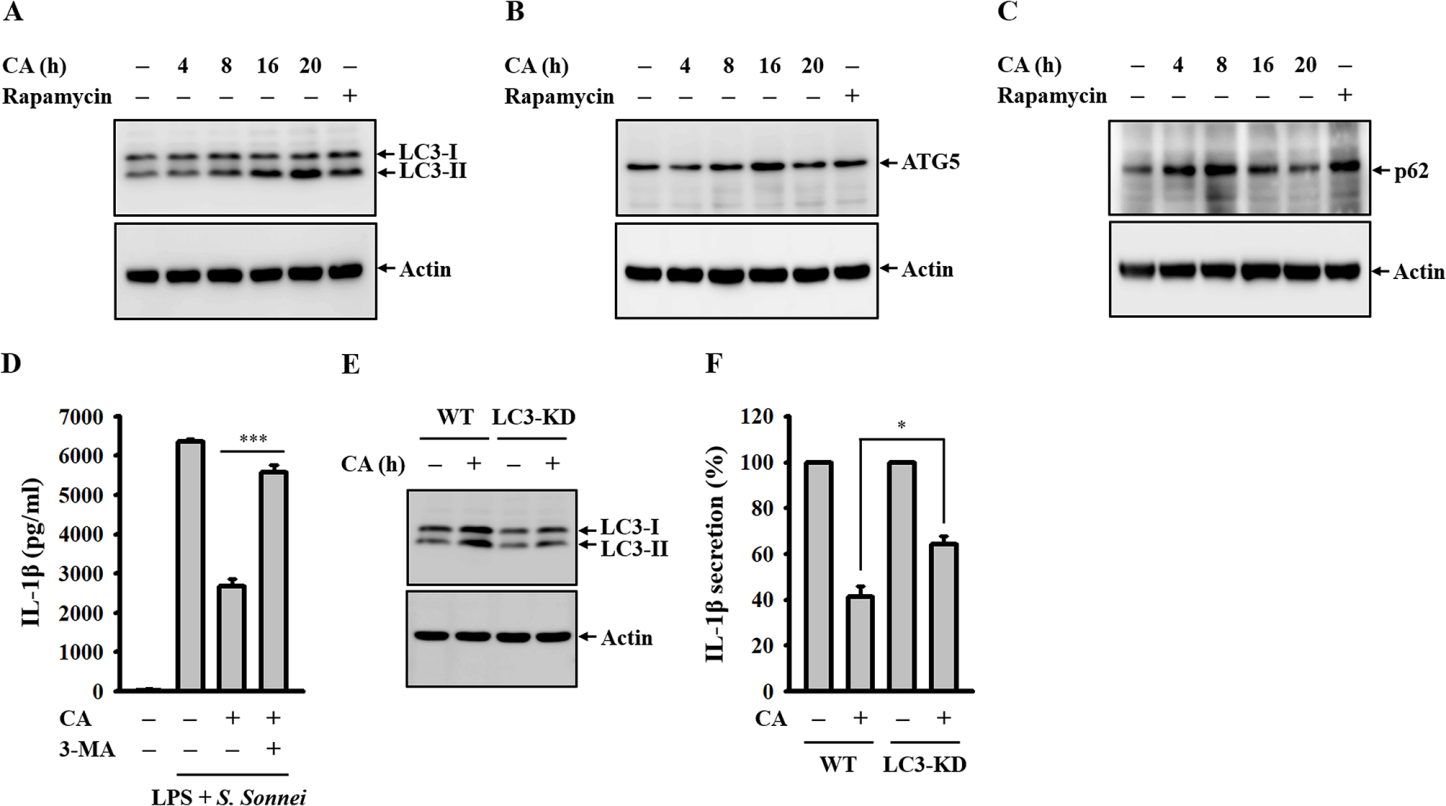
Role of autophagy in CA-mediated IL-1β inhibition in *S. sonnei*-infected macrophages. (**A-C**) J774A.1 macrophages were incubated with 40 µM CA for 4–20 h or 1 µM rapamycin for 4 h. The expression levels of LC3 (**A**), ATG5 (**B**) and p62 (**C**) in the cell lysates were analysed by Western blotting. (**D**) J774A.1 macrophages were primed for 4 h with LPS in the presence or absence of 1 mM 3-MA, followed by incubation with 40 µM CA for 0.5 h before *S. sonnei* infection for an additional 21 h. The detectable levels of IL-1β in the supernatants were analysed by ELISA. (**E**) Wild-type or LC3-knockdown J774A.1 macrophages were incubated with 40 µM CA for 20 h. The expression levels of LC3 in the cell lysates were analysed by Western blotting. (**F**) LPS-primed wild-type or LC3-knockdown J774A.1 macrophages were incubated with 40 µM CA for 0.5 h before *S. sonnei* infection for an additional 21 h. The detectable levels of IL-1β in the supernatants were analysed by ELISA. The Western blotting images are representative of separate experiments. The ELISA data are expressed as the means ± SD of three separate experiments. *p < 0.05 and ***p < 0.001 as indicated

Autophagy has been demonstrated to serve as a self-protective mechanism against IL-1β production mediated by the NLRP3 inflammasome [31]. Previous studies from our lab underscored the significance of autophagy induction as a key mechanism behind the inhibitory effects of small molecules on the NLRP3 inflammasome [25, 32–34]. Consequently, we sought to explore whether CA’s ability to inhibit NLRP3 is linked to autophagy induction. J774A.1 macrophages were treated with CA for 4–20 h or with the autophagy inducer rapamycin for 4 h. Our findings revealed that CA elevated the expression levels of the autophagy markers LC3-II (Fig. 6A), ATG5 (Fig. 6B), and p62 (Fig. 6C), suggesting its role in inducing autophagy in macrophages. To delve into whether CA-mediated autophagy contributes to its inhibitory effect on the NLRP3 inflammasome in *S. sonnei*-infected macrophages, LPS-primed J774A.1 macrophages were treated with CA for 0.5 h in the presence or absence of the autophagy inhibitor 3-MA, followed by infection with *S. sonnei* for an additional 21 h. Notably, 3-MA significantly attenuated CA’s IL-1β inhibitory activity in *S. sonnei*-infected macrophages (Fig. 6D). Furthermore, the IL-1β inhibitory effect of CA diminished in LC3-knockdown J774A.1 macrophages compared to control cells, underscoring the involvement of autophagy in CA-mediated NLRP3 inflammasome inhibition (Fig. 6E and F).

### CA enhances the phagocytosis and bactericidal activity of macrophages against *S. Sonnei*

**Fig. 7:**
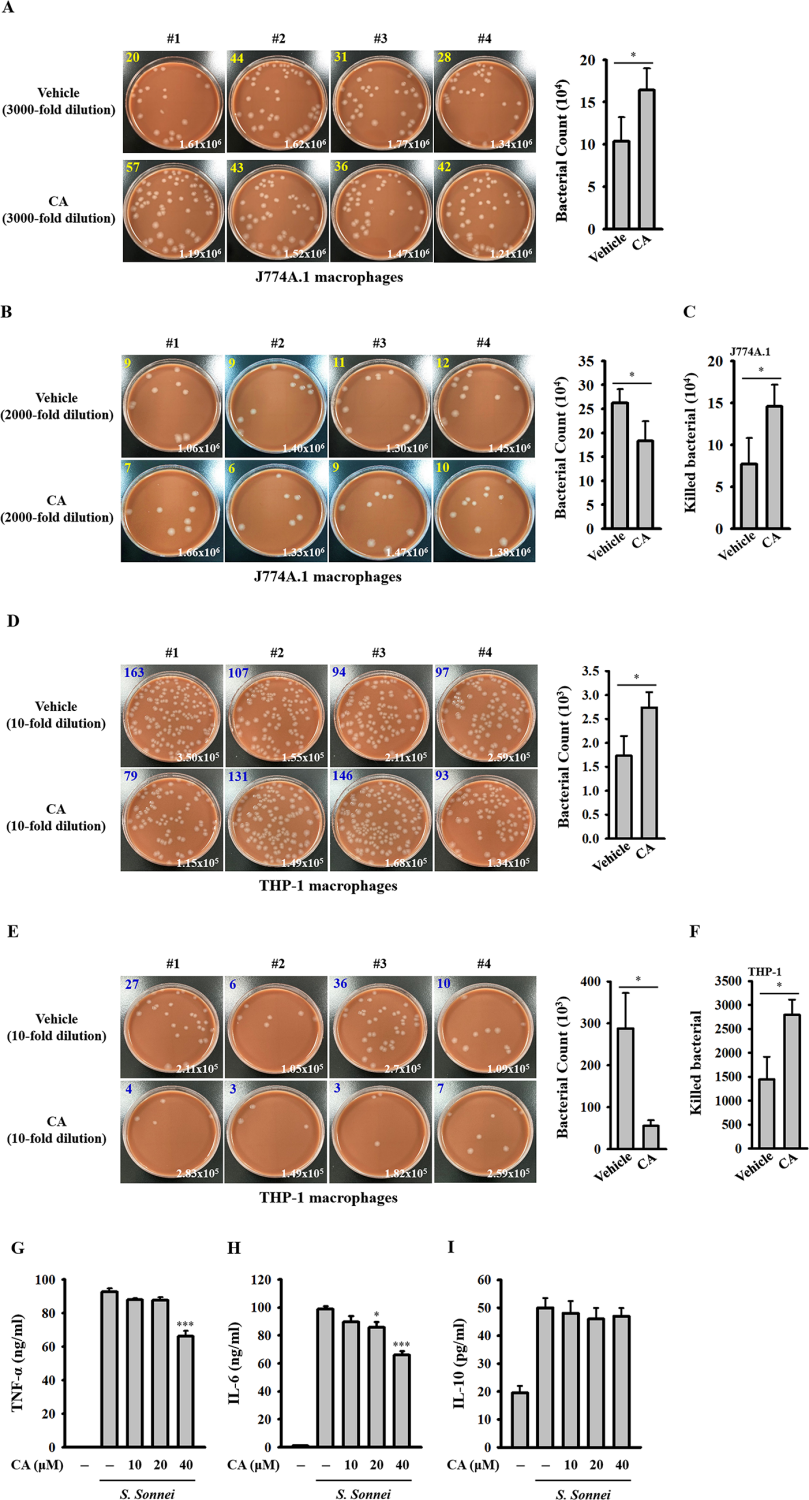
Effect of CA on the phagocytosis and bactericidal activity of macrophages against *S. sonnei*. (**A-F**) J774A.1 macrophages or THP-1 macrophages were pre-treated with CA for 24 h, followed by *S. sonnei* infection for either 15 min (**A, D**) or 20 h (**B, E**). Subsequently, the cells were lysed, and the number of engulfed live *S. sonnei* was determined using the CFU assay. The CFU count and cell number are denoted in the upper left and lower right corners of the images, respectively. To assess bactericidal activity, the number of killed bacteria was calculated by subtracting the CFU count at 20 h from that at 15 min (**C, F**). (**G-I**) J774A.1 macrophages were incubated with CA for 24 h, followed by *S. sonnei* infection for 21 h. The levels of TNF (**G**), IL-6 (**H**) and IL-10 (**I**) in the supernatants were quantified using ELISA. Data represent the means ± SD from four independent experiments (**A-F**) or three independent experiments (**G-I**). Statistical significance was determined by *p < 0.05 and ***p < 0.001 compared to *S. sonnei*-infected macrophages (**G-I**) or as indicated

We conducted further investigation into whether CA could enhance the host’s defense against *S. sonnei*. J774A.1 macrophages were treated with CA for 24 h, followed by *S. sonnei* infection for 15 min. Our findings revealed that the number of engulfed bacteria in CA-treated cells was higher compared to vehicle-treated cells (Fig. 7A), indicating that CA promotes macrophage phagocytosis against *S. sonnei*. To delve deeper into the functional impact of CA treatment on macrophage bactericidal activity against *S. sonnei*, we assessed the number of intracellular live *S. sonnei* cells after 20 h of infection using the CFU assay. It was observed that the number of intracellular live *S. sonnei* cells in CA-treated cells was lower than in vehicle-treated cells (Fig. 7B). By subtracting the 20-hour CFU assay result from the 15-minute CFU assay result, we calculated the number of killed *S. sonnei* cells. Notably, CA treatment significantly enhanced the bactericidal activity of macrophages against *S. sonnei* (Fig. 7C). We extended our investigation of CA’s effects on phagocytosis and bactericidal activity to human THP-1 macrophages under identical experimental conditions as the J774A.1 cells. Our results demonstrated that CA also augmented phagocytosis (Fig. 7D) and bactericidal activity (Fig. 7E and F) of macrophages against *S. sonnei*, indicating a consistent enhancement by CA. Furthermore, we explored whether CA could mitigate the inflammatory response in *S. sonnei*-infected macrophages. J774A.1 macrophages were treated with CA for 24 h, followed by *S. sonnei* infection for 21 h. Our findings revealed that CA reduced TNF (Fig. 7G) and IL-6 (Fig. 7H) secretion in *S. sonnei*-infected macrophages. IL-10 has been shown to steer macrophage polarization towards an immunosuppressive M2 phenotype, which exhibits heightened phagocytic activity [35, 36]. In order to delve into the potential mechanism underlying CA-mediated enhancement of phagocytosis, we examined the influence of CA on IL-10 expression in *S. sonnei*-infected macrophages. Interestingly, while *S. sonnei* infection led to an increase in IL-10 expression in J774A.1 macrophages, CA showed no effect on IL-10 expression (Fig. 7I). Further investigation is required to elucidate the mechanism behind CA-mediated phagocytosis.

## Discussion

As a natural antimicrobial agent, CA impedes bacterial growth primarily by disrupting cell membrane integrity. A recent study illustrated that CA hampers the biosynthesis pathways of phosphatidylglycerol and phosphatidylethanolamine in both *Staphylococcus aureus* and *E. coli* [37]. Research indicates that cinnamon essential oils possess minimum inhibitory concentrations against multidrug-resistant Shigella clinical isolates, including *S. sonnei*, ranging from 0.15 to 1.25 µl/ml. Gas chromatography/mass spectrometry analysis has revealed that cinnamon essential oils contain 84.8% CA. This finding strongly implicates CA as the primary active ingredient responsible for the antibacterial properties of cinnamon essential oils [38]. Furthermore, CA not only inhibits bacterial growth but also exhibits anti-inflammatory effects in bacteria-infected cells. In *Helicobacter pylori*-infected gastric epithelial cells, CA impedes NF-κB activation and reduces IL-8 expression [39]. Moreover, in THP-1 macrophages infected with *Aggregatibacter actinomycetemcomitans*, CA suppresses TNF-α and IL-1β expression by mitigating MAPKs phosphorylation and NF-κB activation [40].

Activation of the NLRP3 inflammasome in response to bacterial infection serves pivotal roles in maintaining homeostasis against invading pathogens. However, excessive production of IL-1β and IL-18 from NLRP3 inflammasome-activated cells can induce tissue damage and elevate the risk of disease development. Research has shown that infection with *Porphyromonas gingivalis* triggers NLRP3 inflammasome activation, fostering a proinflammatory microenvironment conducive to colorectal tumorigenesis [41]. Mounting evidence underscores the crucial involvement of the NLRP3 inflammasome in inflammatory damage induced by pathogenic bacteria, including *Mycobacterium tuberculosis* [42], *Herpes simplex virus-1* [43], *Neisseria gonorrhoeae* [24], and *Klebsiella pneumoniae* [44]. In our previous study, we provided evidence of NLRP3 inflammasome activation in macrophages infected with *S. sonnei* [13]. Inhibiting NLRP3 inflammasome activation in *S. sonnei*-infected macrophages was achieved by reducing mitochondrial ROS production and preserving mitochondrial integrity, highlighting the pivotal role of mitochondrial damage in *S. sonnei*-mediated IL-1β production [13]. Furthermore, our study demonstrated the significant inhibitory effect of CA on NLRP3 inflammasome activation in *S. sonnei*-infected macrophages. Mechanistically, we observed that CA mildly decreased mitochondrial ROS production and mitigated the loss of mitochondrial integrity in *S. sonnei*-infected macrophages. However, since this modest protection of mitochondria did not fully account for the substantial inhibitory effect on the NLRP3 inflammasome, we revealed that CA-induced autophagy partly contributes to its inhibitory action on the NLRP3 inflammasome. The capacity of CA to induce autophagy has been observed in various cell types. For instance, in *A. actinomycetemcomitans*-infected THP-1 macrophages, CA induced autophagy and reduced IL-1β expression [40]. Additionally, CA triggered autophagy in human gastric carcinoma cell lines NCI-N87 and MKN-74, where this autophagic response played a critical role in CA-mediated cell death in gastric carcinoma cells [45]. Furthermore, oral administration of CA (40 mg/kg) for 29 days enhanced the autophagic response in the liver tissue of Wistar rats, leading to improved insulin sensitivity and reduced visceral adiposity [46].

**Fig. 8:**
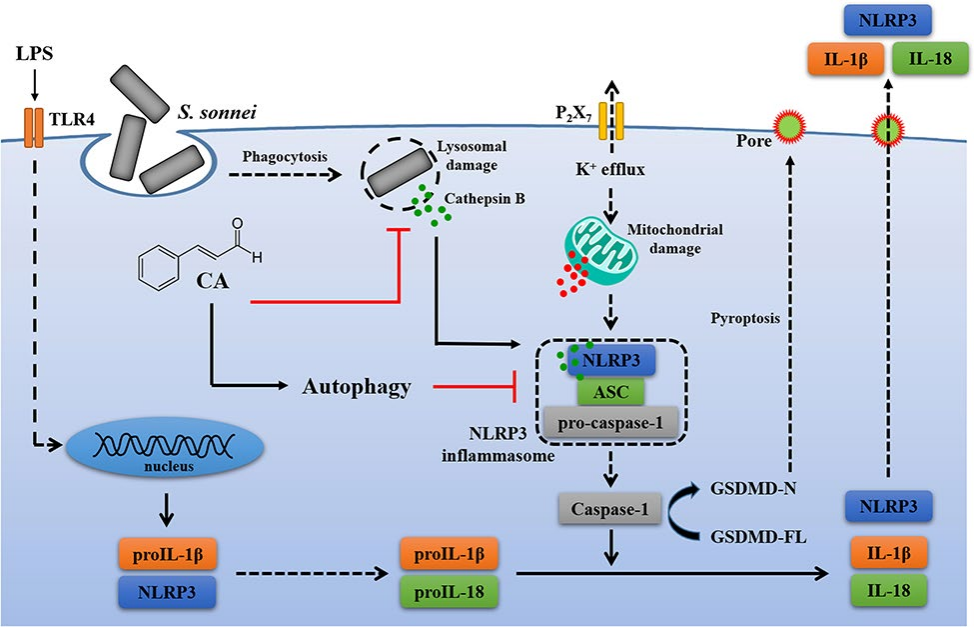
Overview of the demonstrated and putative mechanisms by which CA inhibited the NLRP3 inflammasome in macrophages

While our study marks the first to unveil the inhibitory potential of CA on the NLRP3 inflammasome in *S. sonnei*-infected macrophages, several other experimental models have explored CA’s impact on the NLRP3 inflammasome across diverse contexts. For instance, in mice injected with LPS, CA (at doses of 0.45 or 0.9 mg/kg) significantly attenuated IL-1β levels in peripheral tissues, spleen, and mesenteric lymph nodes [47]. Additionally, in fructose-fed rats, CA (ranging from 20 to 80 mg/kg) mitigated insulin resistance, cardiac hypertrophy, and fibrosis while suppressing NLRP3 inflammasome activation in the heart [23]. In THP-1 macrophages, CA impeded NLRP3 inflammasome activation and downregulated the expression levels of NLRP3, active caspase-1, P2 × 7 receptor, and cathepsin B, evident in the lungs of LPS-injected mice [22]. Moreover, CA (at 10 mg/kg) ameliorated DSS-induced colitis in mice by NLRP3 inflammasome inhibition, potentially attributed to reduced expression levels of miR-21 and miR-155 in the colon [48]. Notably, CA (at 200 mg/kg) significantly lowered IL-1β levels in the peripheral blood of adjuvant arthritis rats, possibly through reduced expression of the succinate receptor GPR91, thereby inhibiting HIF-1α activation [49]. Beyond its anti-inflammatory properties, CA has demonstrated antidepressant-like effects by reversing depression-like behaviors in rats subjected to chronic unpredictable mild stress. The antidepressant mechanism of CA may involve inhibiting NF-κB and the NLRP3 inflammasome in the prefrontal cortex and hippocampus of rats [50]. In our investigation, we found that CA suppressed NLRP3 inflammasome activation in *S. sonnei*-infected macrophages by dampening activation signals while leaving priming signals unaffected. Although CA at 40 µM significantly inhibited caspase-1 activation and IL-1β expression in *S. sonnei*-infected macrophages, it had no effect on the expression levels of NLRP3 or proIL-1β in LPS-activated J774A.1 macrophages. This suggests that CA does not hinder the priming signals of the NLRP3 inflammasome induced by LPS. Furthermore, CA at 40 µM did not impede LPS-induced MAPKs phosphorylation or NF-κB activation, both pivotal in NLRP3 and proIL-1β expression [4]. However, intriguingly, CA at 80 µM or 24 µM significantly curtailed LPS-induced proIL-1β expression in J774A.1 macrophages and THP-1 monocytes, respectively [18]. At 80 µM, CA notably inhibited LPS-mediated proIL-1β expression, partly achieved by suppressing JNK1/2 phosphorylation, known regulators of proIL-1β expression in LPS-activated macrophages [51]. Collectively, CA exhibits bactericidal activity against *S. sonnei* [38] and curbs NRLP3 inflammasome activation triggered by *S. sonnei* infection. These findings underscore CA’s potential as a promising nutraceutical for the prevention and alleviation of bacillary dysentery (Fig. 8).

While excessive or uncontrolled inflammation can inflict tissue damage and contribute to various host diseases, appropriately triggered inflammation by the host immune system is indispensable. It serves to recruit immune cells to infection sites, bolster pathogen clearance, and initiate adaptive immune responses, all pivotal for microbial pathogen defense [52]. Therefore, while the mitigation of inflammation and the prevention of inflammatory cell death in infected macrophages through CA may initially seem advantageous for alleviating tissue damage and inflammation-related symptoms, it could inadvertently create a favorable milieu for the survival and dissemination of *S. sonnei*. Striking a balance between controlling inflammation and effectively combating bacterial infection is paramount in devising therapeutic strategies against *S. sonnei*. While our findings illustrate the potential of CA in enhancing macrophage-mediated defenses against *S. sonnei*, it is essential to delve deeper into CA’s in vivo efficacy against *S. sonnei* infection, as well as its inhibitory effect on the NLRP3 inflammasome, utilizing a mouse infection model. This further exploration is imperative to fully grasp the extent of CA’s impact on *S. sonnei* infection.

## Data Availability

The data used to support the findings of this study are available from the corresponding author upon request.
